# Role of ^18^F-fluorodeoxyglucose (FDG) and ^18^F-2-fluorodeoxy sorbitol (FDS) in autoimmune hypophysitis: a case report

**DOI:** 10.1186/s12902-020-00567-8

**Published:** 2020-06-09

**Authors:** Ziren Kong, Yu Wang, Wenbin Ma, Xin Cheng

**Affiliations:** 1 Department of Neurosurgery, Peking Union Medical College Hospital, Chinese Academy of Medical Sciences and Peking Union Medical College, No.1 Shuaifuyuan Wangfujing Dongcheng District, Beijing, China; 2 Department of Nuclear Medicine, Peking Union Medical College Hospital, Chinese Academy of Medical Sciences and Peking Union Medical College, No.1 Shuaifuyuan Wangfujing Dongcheng District, Beijing, China

**Keywords:** Autoimmune hypophysitis, PET, FDG, FDS, Case report

## Abstract

**Background:**

Autoimmune hypophysitis is a rare disease characterized by the infiltration of lymphocytic cells into the pituitary gland. ^18^F-fluorodeoxyglucose (FDG) and ^18^F-2-fluorodeoxy sorbitol (FDS) positron emission tomography (PET) are well-established and emerging techniques, respectively, which may aid in the diagnosis and classification of autoimmune hypophysitis.

**Case presentation:**

Here, we report a 40-year-old female diagnosed with central diabetes insipidus and multiple pituitary hormone deficiencies, and MRI revealed homogeneous signals in the pituitary gland as well as thickened in the pituitary stalk. FDG PET localized the pituitary and pituitary stalk lesions and displayed an SUVmax of 5.5. FDS, a sensitive radiotracer for bacterial infections but remains unproven under aseptic inflammation, also demonstrated elevated radioactivity, with an SUVmax of 1.1 at 30 min and 0.73 at 120 min. Transnasal biopsy suggested a diagnosis of autoimmune hypophysitis, and the patient displayed radiological and clinical improvement after treatment with glucocorticoids and hormone replacement.

**Conclusions:**

Autoimmune hypophysitis can display elevated FDG uptake, which aids in the localization of the lesions. In addition to revealing bacterial infection specifically, FDS can also accumulate under autoimmune conditions, suggesting that it could serve as a potential radiotracer for both bacterial and aseptic inflammation.

**Trial registration:**

The patient was enrolled in study NCT02450942 (clinicaltrials.gov, Registered May 21, 2015).

## Background

Autoimmune hypophysitis, also known as lymphocytic hypophysitis, is characterized by the infiltration of lymphocytic cells into the pituitary gland due to an autoimmune etiology that leads to pituitary dysfunction and occurs in pituitary patients with an incidence ranging from 0.24 to 0.87% [1–4]. Positron emission tomography (PET) is a well-established molecular imaging technique that assesses cellular metabolism using radiolabeled substrates [5]. ^18^F-fluorodeoxyglucose (FDG) is the most widely used radiotracer to aid in the localization and diagnosis of diseases. However, altered glucose metabolism can occur in multiple circumstances [6–8], and FDG alone may be insufficient to differentiate neoplastic, infectious, or autoimmune diseases. ^18^F-2-fluorodeoxy sorbitol (FDS) is an analog of sorbitol that can be taken up by Enterobacteriaceae [9, 10], leading to its use as a sensitive radiotracer for infections. However, the utility of FDS under aseptic inflammation faces controversies [10, 11]. This article reports a case of pathologically diagnosed autoimmune hypophysitis that displayed FDG and FDS activity, which highlights the significance of PET in disease localization and the potentialities of FDS in sterile inflammation.

## Case presentation

**Fig. 1:**
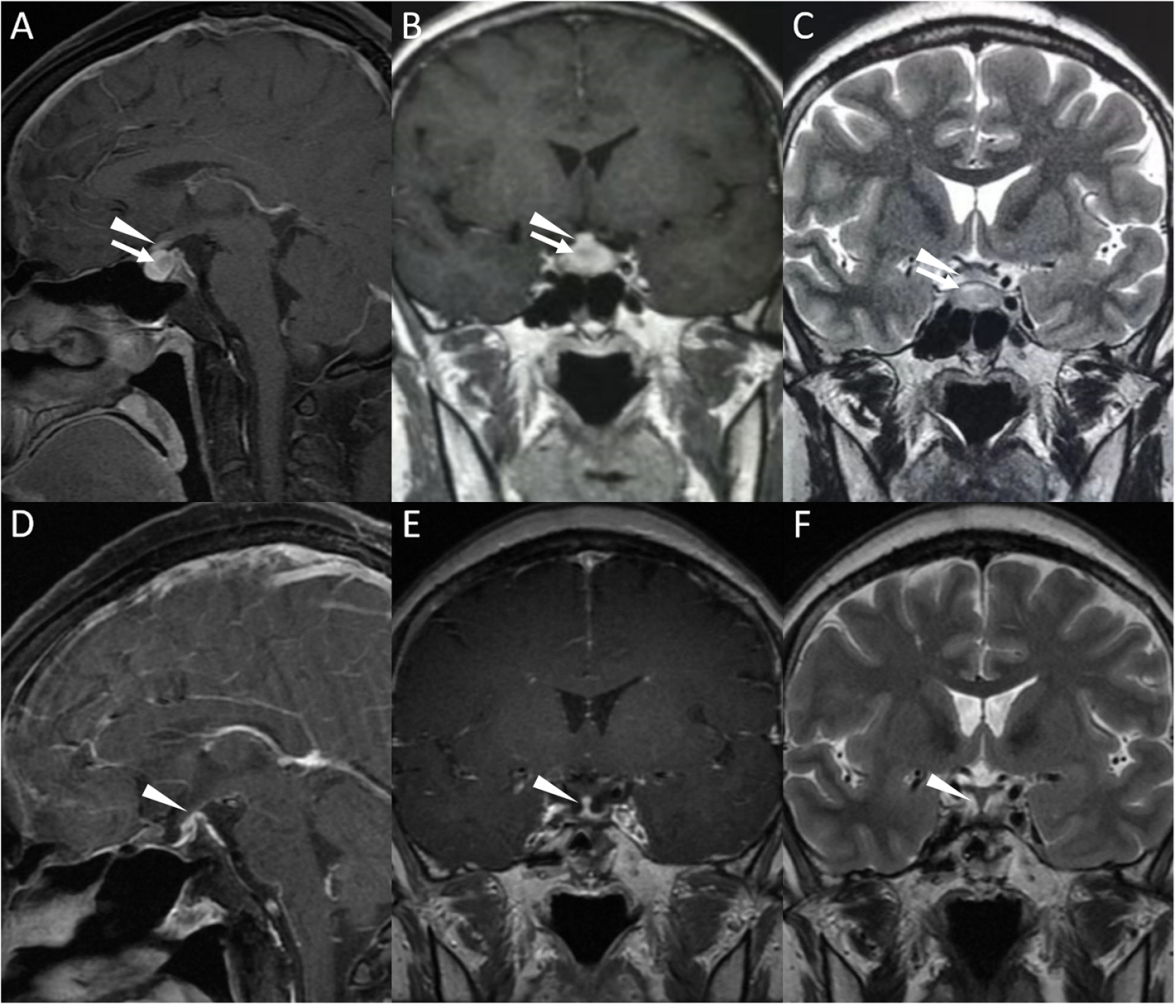
Contrast-enhanced T1-weighted and T2-weighted MR images of the lesions. **a-b**. Pituitary with a size of 18.6 mm × 8.2 mm × 9.9 mm displayed a contrast-enhanced signal, and a lesion with a size of 6.5 mm × 5.2 mm × 4.6 mm and a relative hypointense contrast-enhanced signal was located (arrow noted). The pituitary stalk with a size of 5.1 mm × 1.7 mm showed an isointense contrast-enhanced signal (arrowhead noted) compared with the normal pituitary stalk. **c**. The pituitary lesion presented a hyperintense T2-weighted signal (arrow noted), and the thickened pituitary stalk exhibited an isointense T2-weighted signal in comparison with the normal pituitary. **d-f**. Contrast-enhanced T1-weighted and T2-weighted MR images of the lesions 3 years after surgery. The pituitary displayed postsurgical changes with no significant hypointense contrast-enhanced signal or hyperintense T2-weighted signal, and the pituitary stalk thickness was reduced (3.0 mm × 2.0 mm, arrowhead noted) compared with pretreatment MRI

**Fig. 2:**
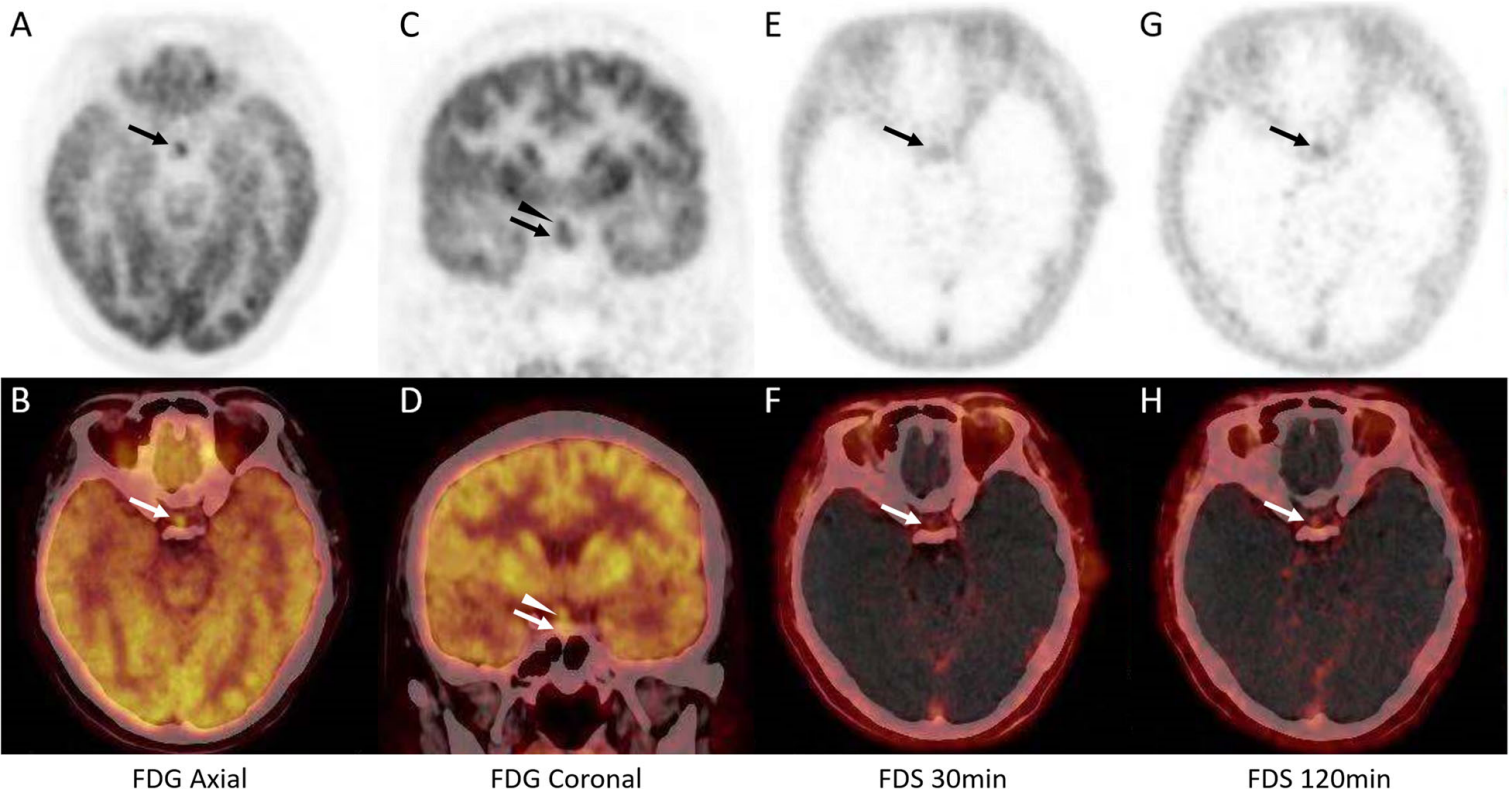
FDG and FDS activity of the lesions. **a-d**. Both the pituitary and pituitary stalk lesions displayed FDG activity with an SUVmax of 5.5 after 40 min of radiotracer injection. **e-f**. Both lesions displayed FDS activity with a SUVmax of 1.1 at 30 min after FDS injection; in comparison, the SUVmax of the normal brain tissue was 0.15, and the T/N ratio was 7.3. **g-h**. The lesions remained FDS active after 120 min of FDS administration with a SUVmax of 0.73; the SUVmax of the normal brain and the T/N ratio were 0.08 and 9.1, respectively

A 40-year-old female with a 4-month history of polydipsia, polyuria, headache, menstrual disorder and fatigue was admitted to the hospital. Examinations revealed a urine osmolality of 63 mOsm/kg when plasma osmolality reached 307 mOsm/kg and serum sodium was 148 mmol/L, and deficiencies of adrenocorticotropic hormone, thyroid-stimulating hormone and gonadotropins. Magnetic resonance imaging (MRI) revealed homogeneous signals in the pituitary gland as well as thickening of the pituitary stalk (Fig. 1), which supported a diagnosis of central diabetes insipidus. FDG PET was performed with a dose of 5.55 MBq (0.15 mCi) per kilogram of body weight to localize the pituitary and/or the pituitary stalk lesions and revealed radioactivity at both sites (Fig. 2). However, the elevated FDG uptake was inadequate for an etiological diagnosis, and FDS (5.55 MBq/kg) was utilized to further explore the nature of the disease. The lesions also showed increased FDS uptake at 30 min and 120 min after FDS injection (Fig. 2), both of which were significantly higher than normal brain uptake. The patient was suspected to have autoimmune or infectious pituitary inflammation based on FDG and FDS activity, and both tracers also excluded possible extracranial involvement.

Transnasal biopsy was performed for a definitive diagnosis. A lesion was found locating at posterior pituitary, while the anterior pituitary remained normal. Histopathology of the posterior pituitary lesion revealed lymphocyte infiltration and fibrosis of the pituitary gland, while no evidence of malignant elements or infection was seen, which indicated a diagnosis of autoimmune hypophysitis. The patient was then treated with oral prednisone (starting dosage of 40 mg/day, gradually decreased every 3 weeks to a maintenance dosage of 10 mg/day), desmopressin acetate (0.2 mg/8 h) and levothyroxine (50 μg/day) and showed an improvement in clinical symptoms and on radiological examination (Fig. 1). However, hypopituitarism remained stable and hormonal replacement was consistently needed.

## Discussion and conclusions

This case reports the FDG and FDS activity in a patient with autoimmune hypophysitis, with the results suggesting the role of FDG PET in differential diagnosis and disease classification as well as the potential application of FDS PET. Autoimmune hypophysitis, according to its infiltrating range, can be classified as lymphocytic adenohypophysitis (involving the anterior pituitary but not the infundibulum or posterior pituitary), lymphocytic infundibuloneurophypophysitis (infiltrating the infundibulum or posterior pituitary or pituitary stalk without anterior pituitary involvement) or lymphocytic panhypophysitis (infiltrating both the anterior pituitary and infundibulum or posterior pituitary or pituitary stalk). In addition to obtaining tissue for definitive diagnosis and classification, the clinical and neuroradiological characteristics of autoimmune hypophysitis together with their improvement during treatment can establish a clinical diagnosis of autoimmune hypophysitis if alternative pituitary diseases and other causes can be excluded [4]. In our case, the MRI results showed abnormal signals of a pituitary lesion and a thickened pituitary stalk, but the signals from the pituitary lesion and thickened pituitary stalk were inconsistent, which caused difficulties in narrowing down the clinical characteristics into a diagnosis of autoimmune hypophysitis. Moreover, alternative disease possibilities such as pituitary abscess, central nervous system germ cell tumor or Langerhans cell histiocytosis needed to be excluded. Thus, metabolic imaging was applied to further investigate the nature and relationship of the lesions.

**Fig. 3:**
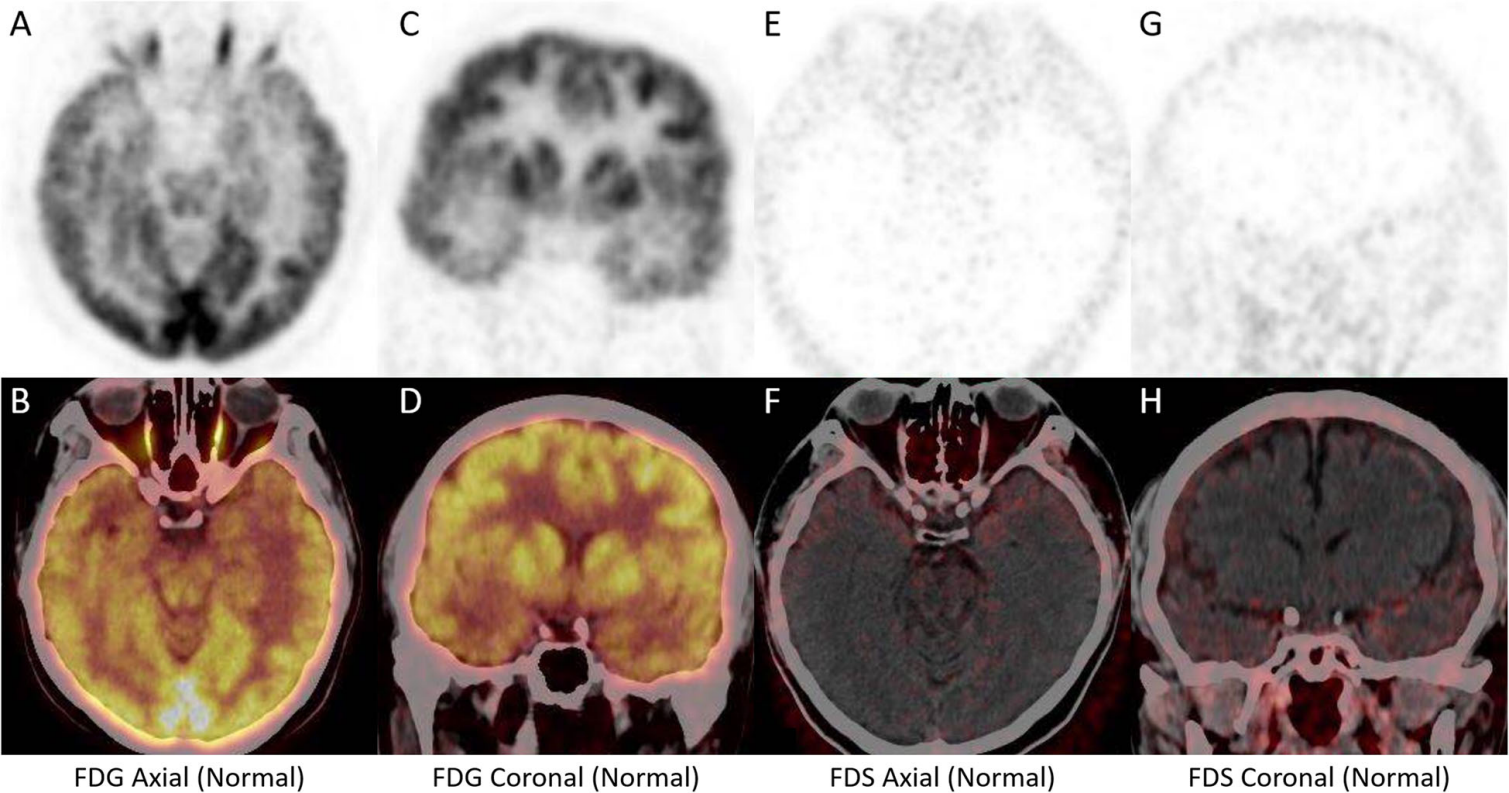
FDG and FDS activity in a patient with a normal pituitary. **a-d**. Both the pituitary and pituitary stalks did not display FDG activity. **e-h**. FDS was also not absorbed in normal pituitary and pituitary stalks

Although the role of FDG PET is somewhat limited by the high background activity when evaluating central nervous system diseases, it provides considerable information for the evaluation of pituitary diseases since the normal pituitary gland does not typically take up FDG (Fig. 3) [12]. The usefulness of FDG PET has been demonstrated in neoplastic diseases such as pituitary adenoma, Langerhans cell histiocytosis and nonneoplastic disorders such as primary hypothyroidism [13–15]. However, only one previous case report described that primary hypophysitis had moderate uptake of FDG and displayed an SUVmax of 4.7 [16]. Recently, owing to the development of immunotherapy, immunotherapy-induced hypophysitis has also been reported [17]. Both anti-cytotoxic T-lymphocyte antigen 4 (CTLA4) and anti-programmed cell death protein 1 (PD1) treatments can result in secondary hypophysitis [18–20] and display elevated FDG radioactivity. In addition, a normalization of FDG uptake can also be observed after hypophysitis treatment [18]. In the present case, mild FDG activity was located in both the pituitary and pituitary stalk lesions, providing substantial evidence for disease classification.

FDS can be synthesized through a three-step automated reaction from ^18^F ions or reduced from FDG with NaBH_4_ [9, 11] and shares a similar structure with FDG. FDS can be rapidly eliminated from organs and blood through the renal-urinary system [9], and due to its favorable renal kinetics, it is also considered a potential tracer for renal functional imaging [21]. Previously, FDS was reported to be absorbed by Enterobacteriaceae, leading to its use as a promising tracer for bacterial infection [9–11]. In our patient, FDS was initially given to exclude pituitary abscesses that can occur without a clear sign of infection [22], and negative results would further eliminate the possibility of infectious diseases. However, the uptake of FDS under aseptic inflammation faces controversies. Li et al. induced sterile inflammation by 12-O-tetradecanoyl-phorbol-13-acetate (TPA) and displayed increased FDS uptake in animal models [11], while Weinstein et al. inoculated mice with heat-killed *E. coli* which did not demonstrate FDS activity [10]. Thus, the increased radioactivity in our case may be the result of either autoimmune hypophysitis or abscess, and biopsy was performed for a definitive diagnosis. Our results demonstrated that aseptic inflammation can also display elevated FDS uptake, and radioactivity can be seen on both the 30 min and 120 min scans, which excluded blood pool effects. To the best of our knowledge, this is the first report of FDS radioactivity in autoimmune conditions in humans, suggesting FDS as a potential radiotracer for both bacterial and aseptic inflammation. Nevertheless, the diagnostic role and underlying mechanism of FDS in inflammatory circumstances need to be further investigated, and a comparison of diagnostic performances with widely applied radiotracers (e.g., FDG) is required.

In conclusion, autoimmune hypophysitis is a rare disease whose diagnosis and classification remain difficult. FDG and FDS PET may provide additional radiological information to aid in the localization and diagnosis of disease. In addition to specifically revealing bacterial infection, FDS can also accumulate under autoimmune inflammation, indicating another potential application.

## Data Availability

The datasets used during the current study are available on request from the corresponding author.

## Abbreviations

CTLA4: Cytotoxic T-lymphocyte antigen 4
FDG: ^18^F-fluorodeoxyglucose
FDS: ^18^F-2-fluorodeoxy sorbitol
MRI: Magnetic resonance imaging
PD1: Programmed cell death protein 1
PET: Positron emission tomography
TPA: 12-O-tetradecanoyl-phorbol-13-acetate
